# Lung electrical impedance tomography during positioning, weaning and chest physiotherapy in mechanically ventilated critically ill patients: a narrative review

**DOI:** 10.1186/s13613-025-01526-z

**Published:** 2025-08-29

**Authors:** Sam Bayat, Claude Guérin, Bruno Louis, Nicolas Terzi

**Affiliations:** 1 Université Grenoble Alpes, STROBE INSERM UA07, Grenoble, France; 2 Service de Pneumologie et Physiologie, CHU Grenoble-Alpes, Grenoble, France; 3 Faculté de Médecine Lyon Est, Université de Lyon, 8 Avenue Rockefeller, 69008 Lyon, France; 4 Institut Mondor de Recherches Biomédicales INSERM-UPEC U955 CNRS EMR7000, Créteil, France; 5 Médecine Intensive Réanimation, CHU de Rennes, Rennes, France

**Keywords:** Electrical impedance tomography, Clearing airways secretion, Intensive care unit, Lateral position, Positioning, Prone position, Semi-recumbent position, Chest physiotherapy, Weaning

## Abstract

**Background:**

Electrical impedance tomography (EIT) is a non-invasive, radiation free, lung imaging technique of lung ventilation with a low spatial but a high temporal resolution available at the bedside. Lung perfusion, and hence ventilation-to-perfusion ratios, can also be assessed with EIT. Most of the EIT studies in intensive care units (ICU) are dedicated to positive end expiratory pressure selection in patients with acute respiratory distress syndrome receiving invasive mechanical ventilation. This narrative review explores the use of EIT during change in body position, weaning and chest physiotherapy in adult intubated ICU patients.

**Main body:**

EIT findings confirm a better ventilation and the persistence of lung perfusion in the dorsal lung regions in prone as compared to supine position. However, the response of the ventilation distribution to prone is heterogeneous across patients. For the weaning, global inhomogeneity index, end-expiratory lung impedance, absolute ventral-to-dorsal difference of the change in lung impedance and temporal skew of aeration had a good performance to predict spontaneous breathing trial (SBT) failure in some observational studies. Pendelluft that measures the risk of overstretching in dependent lung regions can only be assessed with EIT. It occurs frequently during weaning and is associated with poor patient outcome. However, its performance to predict SBT failure was moderate. Randomized controlled trials comparing SBT techniques did not find a difference in EIT indexes. The effects of other body positions and chest physiotherapy have been less investigated with EIT.

**Conclusion:**

EIT offers the possibility to monitor lung ventilation and perfusion at the bedside and hence to deliver a personalized ventilatory management. Further designed EIT studies coupled with measurement of lung aeration and patient breathing effort are warranted during weaning to check if the technique is useful to clinical outcome. The same is true regarding the optimal use of body position including prone, and of chest physiotherapy in ICU patients.

**Supplementary Information:**

The online version contains supplementary material available at 10.1186/s13613-025-01526-z.

## Introduction

**Fig. 1:**
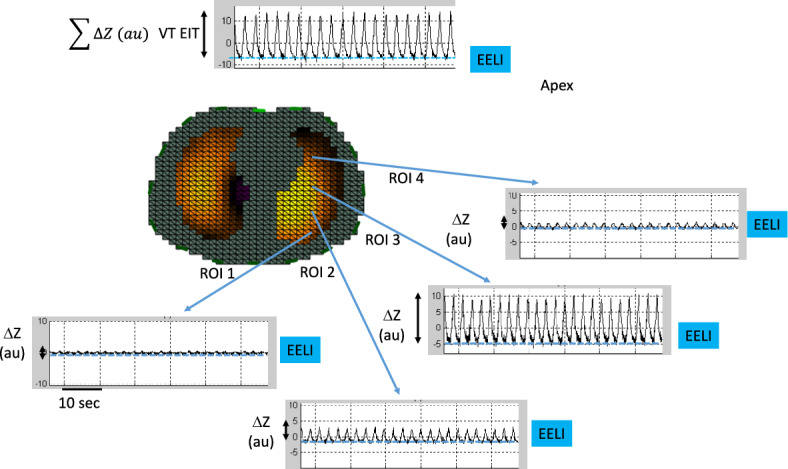
Change in lung impedance (ΔZ) in arbitrary units (au) over time (time scale 10 s) within 4 regions of interest (ROI) arranged as layers Figured out by the blue cubes, in the axial section of the right lung in the supine position. The black double-arrowed vertical lines are the amount of ΔZ in each ROI. The blue broken horizontal lines underline the end expiratory lung impedance (EELI) in each ROI. The tracing on the top is the global ΔZ, which represents the related tidal volume (VT EIT)

Electrical impedance tomography (EIT) is a non-invasive, radiation free, low spatial resolution lung imaging technique that measures ventilation and perfusion distribution. Though lung ventilation can be measured at the bedside with all the devices, this is not yet the case for lung perfusion. EIT principles have been extensively reviewed [1–3]. The map of the electrical impedance obtained by a series of electrodes embedded in a belt surrounding the mid-thorax can be interpreted as a variation of gas volume (Fig. 1) or as a variation of blood flow [4]. It is increasingly used in patients receiving invasive mechanical ventilation in the intensive care unit (ICU) [1, 5]. Various EIT indexes pertaining to lung ventilation can be used. The end-expiratory lung impedance (EELI) reflects end-expiratory lung volume and can be used to assess lung derecruitment. However, EELI is extremely vulnerable to artifacts [6]. Pendelluft defined as asynchronous alveolar ventilation caused by different regional time constants, increases the risk of overdistension in the dependent lung regions, which receive excess ventilation from the non-dependent lung regions during the same inspiration in spontaneous assisted breathing [7] potentially associated with increased respiratory workload and lung overstreching [8]. However, no standardized definition or widely accepted thresholds for clinical significance exists. Recent work suggests a more specific measure [9]. The global inhomogeneity index (GII), the center of ventilation, the anterior-to-posterior impedance ratio, the non-dependent to dependent impedance ratio can assess the heterogeneity of lung ventilation while temporal skew of aeration (TSA) explores the time asynchrony of the ventilation. GII, center of ventilation, pendelluft and TSA are not generally inferred by commercial devices and should be estimated with offline analysis from reconstructed image data. It is important, especially for a reader not very familiar with EIT, to make a distinction across the EIT parameters directly available at the bedside for clinicians, and those which need offline analysis and, hence cannot be immediately used for bedside intervention. The additional Table 1 provides a list of EIT indexes available at the bedside. The offline derived EIT indexes require skills in computation and complex software management.

**Table 1:** Studies on electrical impedance tomography of body positioning in adult intubated ICU patients

# study	First author, year (paper reference)	N Patients Case mix	Age, years (%male)	Mean days intubated	Study design	Position	Primary end-points	Main results	Benefit of EIT assessment and bedside availability of EIT indexes
1	Mezidi, [33]	27ARDS	NA (NA)	NA	Prospective Two centers	Prone	Two PEEP strategies in supine and prone positions	Higher dorsal lung compliance in prone than in supine	Regional lung mechanics assessment in prone position D
2	Dalla Corte, [39]	16 ARDS	57 (50)	2	Prospective Observational Single center	Prone	Effect of prone position on lung ventilation distribution	In prone versus supine redistribution of tidal volume to dependent lung regions, decrease collapse and overdistension	Suggests that prone position is a lung protective strategy A
3	Perier, [42]	14 COVID	54 (4)	9	Prospective Observational Single center	Prone	To describe the effects of prone and supine on lung ventilation distribution in COVID and non COVID	Lung collapse and overdistension minimal at PEEP 12cmH2O	EIT may help titrating PEEP in prone A
4	Perier, [47]	9 COVID	53 (89)	NA	Prospective Observational Single center	Prone	To describe the effects of prone and supine on lung ventilation and perfusion distribution in COVID	Prone positioning redistributed ventilation to dorsal regions with no change in perfusion	Description of the effects of prone on lung ventilation and perfusion D
5	Franchineau, [36]	21 ARDS on ECMO	56 (62)	8	Prospective Observational Single center	Prone	Effect of prone position on optimal PEEP during a decremental PEEP based on overdistension/collapse	Prone position increases dorsal EELI and dorsal ventilation irrespective of Crs improvement	Suggests lung protection in prone during ECMO A
6	Cardinale, [29]	24 COVID	60 (83)	3	Prospective Observational Two centers	Prone	Correlation of dependent lung collapse in supine position to oxygenation response in prone	Linear correlation between dependent lung collapse in supine and oxygenation response to prone	> 13.5%dependent lung collapse has a 94% positive predictive value for improved oxygenation in prone in COVID A
7	Pierrakos, [32]	15 COVID breathing spontaneously	62 (54)	4	Prospective Observational Single center	Prone	Effect of prone on inhomogeneity of aeration and recruitment of collapsed lung tissue	Decreased inhomogeneity, increased aeration, and improved dorsal compliance despite no significant changes in oxygenation in prone	Monitoring of lung aeration in prone B
8	Zarantonello, [30]	30 COVID	62 (73)	0	Prospective Observational Single center	Prone	Oxygenation increase with prone positioning is secondary to the improvement of ventilation-perfusion matching	Increased ventilation-perfusion matching with early prone: 90 min after pronation, first session the day of intubation	Elucidates the mechanism of oxygenation improvement with early pronation in COVID B
9	Dos Santos Rocha, [41]	15 COVID intubated and 13 COVID under non-invasive ventilation	65 (68)	NA	Prospective Observational Single center	Prone	Comparison of lung ventilation distribution in prone in intubated versus non-intubated COVID	Lung ventilation increased in dorsal lung regions in intubated but does not change in non-intubated patients. Oxygenation improved similarly in both groups in prone	Differences between invasive and non-invasive mechanical ventilation regarding the effects of prone on lung ventilation distribution. A
10	Fossali, [34]	21 COVID	67 (81)	1	Prospective Single center	Prone	Effects of prone versus supine position on lung protection and ventilation/perfusion distribution	Net lung recruitment and increase in lung ventilation, less atelectrauma in prone. Dead space lower in prone	EIT and CT data consistent on lung recruitment and protection D
11	Otahal, [43]	10 COVID	64 (70)	3.3	Prospective Cross over Two centers	Prone	Measurement of lung overdistension and collapse during a decremental PEEP trial in supine then in prone position	Larger percentage of overdistension in prone and larger percentage of collapse in supine above PEEP 10 cmH2O	Help to personalize PEEP titration in prone A
12	Wang, [31]	10 ARDS	66 (90)	3.2	Prospective Observational Single center	Prone	Impact of prone position on V/Q distribution	Homogenization of V/Q distribution, improvement in dorsal ventilation, mild increase in dorsal perfusion, better V/Q matching, lower shunt in prone	Elucidate ventilation/perfusion matching in ARDS B
13	Morais, [38]	22 COVID	59 (NA)	4	Prospective Single center	Prone	Changes in Crs after pronation are dependent on PEEP and impact regional ventilation	Ventilation increased progressively in non-dependent zones over the decrease of PEEP. Prone position might cause heterogeneous ventilation distribution in patients with increased Crs after pronation (combination of dorsal recruitment and ventral atelectasis)	Describe patterns of response to PEEP in prone depending on Crs A
14	Yuan, [49]	57 ARDS 33 early and 24 persistent (> 7 days)	71 (65)	3	Prospective Observational Single center	Prone	Comparison in changes in ventilation and perfusion distribution after proning in early versus persistent ARDS	Prone position decreased V/Q mismatch in early ARDS but increased V/Q mismatch in persistent ARDS	Distinct effects of prone on V/Q matching according to ARDS stage B
15	Taenaka, [44]	43 COVID	73 (67)	NA	Prospective Observational Single center	Prone	Impact of low or high PEEP and body position on lung ventilation distribution according to lung recruitability	In high recruiters dependent silent spaces decreased at high PEEP in supine and prone. In low recruiters low PEEP in prone reduced dependent and non-dependent silent spaces	Help to personalize PEEP titration in prone taking into account lung recruitability A
16	Lan, [35]	58 ARDS	62 (81)	2	Prospective Randomized controlled trial Single Center	Prone	Lung ventilation distribution in prone + recruitment manoeuvre versus prone alone	Lung ventilation distribution towards dorsal regions is greater in prone + lung recruitment manoeuvre than in prone alone	Useful to optimize lung recruitment manoeuvre in prone position A
17	Yang, [48]	14 ARDS and 15 COVID	65 (72)	8	Prospective Observational Two centres	Prone	Early impact of pronation on Deadspace, shunt, and ventilation/perfusion match	In ARDS improvement in V/Q matching in prone. In COVID decrease in dead space, increase in shunting, increase in dorsal and decrease in ventral V/Q match in prone (overall reduction in V/Q match in COVID)	Elucidate ventilation/perfusion matching across ARDS etiologies D
18	Yang, [50]	25 ARDS Phenotyped based on focal versus non-focal on lung CT and D-dimer levels	66 (72)	NA	Prospective Observational Single center	Prone	Comparison of the effect of prone on V/Q matching ARDS phenotypes	Non-focal and high D-dimer ARDS need longer prone to improve V/Q matching than the focal and low D-dimer patients	Distinct effects of prone on V/Q matching depending on lung morphology and D-dimer B
19	Wang, [107]	18 COVID	76 (67)	1	Prospective Observational Single center	Prone	Changes in V/Q over time during prone session	V/Q matching increased over time in prone but decreased back to supine	Describes the time course of V/Q matching in prone and after pronation B
20	Bein, [108]	7 ARDS (6 trauma)	54 (100)	NA	Prospective Observational Single center	Lateral	Assessing regional lung ventilation during stepwise lateral posture on a kinetic bed	No significant change in lung ventilation distribution	Understanding and guiding posture therapy A
21	Mlček, [109]	5 COVID	67 (40)	3	Prospective Observational Single center	Lateral	Targeting lateral position based on chest-X ray (less aerated lung up, more aerated lung down) and optimal PEEP (minimization of overdistension and collapse)	Targeted lateral position associated with minimal overdistension and collapse versus supine	Targeted lateral positioning with bedside personalized PEEP reduces overdistension and collapse A
22	Roldan, [54]	15 COVID	53 (93)	0.8	Prospective Observational Single center	Lateral	Effect of sequential lateral position as a potential manoeuver of lung recruitment	Lateral position increased EELI, decreased ventral compliance and increased ventral compliance as compared to supine position	Sequential lateral position improves lung ventilation distribution B
23	Huerta, [110]	32 acute respiratory failure (47% COVID)	62 (72)	6	Prospective Observational Single center	Lateral	Effects of postural repositioning as used during routine nursing practice on lung ventilation distribution	EELI increased in dependent and non-dependent lung during lateral position. Regional ventilation decreased in non-dependent and increased in dependent lung. Regional perfusion decreased in dependent lung	Lateralisation does not decrease lung volume in the dependent lung B
24	Marrazzo, [63]	12 COVID	65 (83)	3	Prospective Observational Single center	Semi-recumbent	Difference between PEEP titrated with EIT in supine-flat and semi-recumbent positions	PEEP and global inhomogeneity index lower in semi-recumbent than in supine	Semi-recumbent position associated with more homogenous ventilation compared to supine B
25	Chen, [62]	12 ARDS 5 COVID	64 (53)	1	Prospective Observational Single center	Semi-recumbent	Effect of sitting position at 70 degrees on lung ventilation distribution	In sitting versus supine dorsal ventilation increased and ventral ventilation decreased, ventral change in EELI decreased and dorsal change in EELI increased	Optimizing the sitting position angulation A

Definition of abbreviations: ICU intensive care unit, ARDS acute respiratory distress syndrome, Crs compliance of the respiratory system, CT computed tomography, ECMO extracorporeal membrane oxygenation, EELI end-expiratory lung impedance, EIT electrical impedance tomography, NA not available, PEEP positive end-expiratory pressure, V/Q lung ventilation to perfusion ratio. The assessment of bedside availability of EIT indexes is made according to the following code: A. All indexes are available at the bedside, B most indexes are available at the bedside, C most indexes are not available at the bedside, D no index is available at the bedside

EIT is a complementary tool to other methods exploring important physiological phenomena at the bedside in the setting of ICU patients under invasive mechanical ventilation. One is lung ultrasound [10]. Both EIT and lung ultrasound are non-invasive but differ in several aspects. EIT measures lung ventilation when lung ultrasound explores lung aeration. EIT is better suited for a monitoring purpose due to the intermittent nature of the lung ultrasound assessment. Lung ultrasound findings are dependent on operator experience, location of the probe, and quality of the tissues transparency [11]. Because intubated ICU patients engaged in the weaning process do breathe spontaneously with or without ventilator assistance the measurement of breathing effort is important. This can be done by measuring esophageal pressure [12] or using diaphragm ultrasound [13, 14]. Combination of EIT, lung and diaphragm ultrasound and esophageal pressure (by evaluating, for example, local compliances) has been employed in some studies, which are discussed below.

Recent reviews have described in details the EIT technology [3, 15, 16], the EIT indexes used to assess lung ventilation and lung perfusion [2], and the application of EIT in the ICU [5, 17]. The main field of application focused on acute respiratory distress syndrome (ARDS). The goals were to address with the EIT the basic pathophysiological concepts stemming from lung CT scan investigations [18–20], and to monitor the safety of mechanical ventilation in ARDS patients [6, 21]. Of notice, EIT was used to help the selection of Positive End Expiratory Pressure (PEEP) in this setting [21, 22]. However, there are areas in intubated ICU patients that can benefit from EIT monitoring, namely body positioning, weaning from mechanical ventilation, and chest physiotherapy. The EIT studies in these areas are increasing and it becomes timely to summarize them. We have performed a narrative review on EIT studies in adult patients intubated and mechanically ventilated in ICU covering the body positioning, the weaning and the chest physiotherapy. A Pubmed search was done in each of these fields, whose strategy is provided in the additional file 2. The articles are summarized in Tables 1, 2 and 3. In each of them we also assessed the bedside availability of the EIT indexes used in the studies.

**Table 2:** Studies on electrical impedance tomography during weaning from mechanical ventilation in adult intubated ICU patients

# Study	First author, year (paper reference)	Patients Case mix N (1st SBT)	Age, years (%male)	Mean days intubated	Study design	Part of the weaning investigated	Primary end-points	Main results	Benefit of EIT assessment and bedside availability of EIT indexes
1	Bickenbach, [80]	Mixed surgical medical Prolonged weaning 31 (0)	73 (51.6)	12.4	Prospective Observational Single center	SBT: T-piece	Prediction of SBT failure from EIT	Increase in GII and RVD during T-piece from PS pre-SBT	Association between GII and SBT failure D
2	Zhao, [89]	Medical Prolonged weaning 30 (30)	68 (60)	NA	Prospective Observational Single center	SBT: ATC100%– 70%PEEP5 cmH2O versus CPAP 5–7.5cmH2O	Comparison of intensity of SBT techniques and prediction of SBT failure from EIT	Higher intensity of ATC or CPAP associated with higher EELI Redistribution of intra-tidal gas distribution towards dorsal regions with lower intensity associated with higher weaning success	Association between ventilation distribution and weaning success A
3	Guérin, [90]	Medical 20 (16)	60 75	8.6	Prospective Interventional, controlled, randomized Single center	SBT: PS7 cmH2O/4 cmH2O/ATC0% versus PS0 cmH2O/4 cmH2O/ATC100%	Comparison of SBT techniques on breathing power	Higher breathing power with ATC	No difference in ventilation distribution between SBT B
4	Longhini, [81]	Mostly medical At risk for extubation failure 78 (78)	NA NA	NA	Prospective Observational Multicentre	SBT: CPAP 2 cmH2O	Pathophysiology and prediction of SBT and extubation failure from EIT	Greater reduction in change in EELI higher GII in SBT failure than in SBT success	Moderate performance of EIT to predict SBT and extubation failure B
5	Lima, [86]	Mixed medical surgical 42 (42)	68 (57)	3	Prospective Observational Single center	SBT: PS or T-piece not randomized	Pathophysiology and prediction of SBT and extubation failure from EIT	No change in EELI between PS failure or PS success, lower EELI in T-piece failure than success	Good performance of change in EELI to predict T-piece success A
6	Coppadoro, [8]	Mixed medical surgical 20 (20)	65 (55)	3	Prospective Observational Single center	PS reduction from 12 to 2cmH2O	Pathophysiological role of pendelluft during PS reduction	Pendelluft observed with PS reduction in 8 (40%) patients, moving gas from ventral to dorsal lung and associated with increase in EtCO2 and respiratory rate	Pendelluft is a EIT index candidate to monitor D
7	Moon, [79]	Medical 40 (40)	69 (72.5)	4.5	Prospective Observational Single center	SBT: T-piece	Predicting SBT failure/success from EIT taking into account diaphragm dysfunction	EIT indexes worsened during SBT failure	Performance for SBT failure: good for temporal skew of aeration and GII and moderate for change in EELI C
8	Li, [87]	Post-operative abdominal surgery 32 (32)	60 (69)	2.6	Retrospective Observational Single center	SBT: low PS + PEEP ≤ 5 cmH2O	Predicting SBT failure/success	At 15 and 30 min SBT EELI and RVD had area under curve > 0.80 to predict SBT failure	EIT can predict SBT failure in patients with delayed extubation after abdominal surgery C
9	Wang G, [83]	Mostly post-operative 53 (53)	62 (62)	7	Prospective Nested case–control Single center	SBT: T-piece	Predicting SBT and weaning failure/success from EIT	Impedance change greater before SBT and in the middle lung during SBT in weaning success than failure	Moderate performance of GII before SBT and impedance change in middle lung during SBT to predict weaning failure B
10	Wang D, [111]	Mostly medical 60 (60)	69 66	NA	Prospective Observational Single center	SBT: low PS + PEEP3-5 cmH2O	Assessment of pendelluft in the weaning process	Pendelluft was higher in the weaning failure than success	Moderate performance of pendelluft to predict weaning failure D
11	Jousselin, [84]	Medical One risk factor for extubation failure 40 (NA)	59 (72)	10	Prospective Observational Single center	Extubation after a successful SBT (T-piece)	To compare lung ventilation (EIT) before and up to 6 h after extubation	Between extubation failure and success surface for ventilation was lower before and after extubation and GII was higher after extubation	Performance of EIT to predict extubation failure was moderate C
12	Liu, [74]	Mixed medical surgical Difficult to wean 108 (24)	64 (72.2)	8	Retrospective Observational Single center	SBT: T-piece (108)	The association of pendelluft during SBT to mortality	Pendelluft during SBT in 70% of patients and significant independent risk factor for mortality at day 28	Pendelluft is a EIT index candidate to monitor D
13	Bosch-Comte, [91]	Medical 43 (43)	59 (56)	7	Prospective Interventional, controlled, randomized Single center	SBT: T-piece versus PS 8cmH2O + PEEP5cmH2O	Changes in lung ventilation induced by SBT in relation with breathing effort	Reduction of EELI occurred during SBT but was not significantly different between SBT techniques. No relation with breathing effort	EIT did not find difference in EELI between T-piece and low PS A
14	Wisse, [82]	Mostly medical 23 (23)	63 (61)	11	Prospective Observational Single center	SBT: T-piece	To predict SBT failure from EIT	Between SBT failure and success, EELI did not differ, GII was higher	Inhomogeneity in lung ventilation can predict SBT failure C
15	Wang P, [92]	Medical Prolonged mechanical ventilation 24 (NA)	68 (62.5)	37.8	Prospective Interventional, controlled, randomized Single center	SBT: T-Piece, ATC, CPAP	To study lung ventilation in different SBT techniques	No difference in EIT indexes between the three SBT techniques Interpatient variability of EIT indexes	Personalisation of SBT with EIT Pendelluft did not change during any SBT technique C
16	Coudroy R, [30]	Medical Risk factors for extubation failure 25 (22)	73 (68)	8	Secondary analysis Interventional, controlled, randomized Multicentre	SBT: T-Piece, PS8cmH2O + PEEP0cmH2O	To study lung derecruitment (change in EELI) during SBT	Significant reduction of EELI after SBT greater with T-piece than PS After restoring mechanical ventilation EELI returned to pre-SBT	EIT can demonstrate the loss of lung ventilation during SBT A
17	Phoophiboon, [85]	Mixed medical surgical 98 (NA)	58 (65)	6	Prospective Observational Multicentre	SBT: T-piece	Prediction of early and successful liberation from mechanical ventilation from EIT recorded during SBT	From the first minutes to the entire SBT duration, the absolute ventral-to-dorsal difference was consistently smaller in liberation success compared to all subgroups of failure	Good performance of absolute ventral-to-dorsal impedance difference during SBT to predict failure of liberation of mechanical ventilation Pendelluft had a poor performance B
18	Da Rosa, 2025	18 (NA)	64 (78)	NA	Prospective Observational Single center	SBT: NA	Feasibility study of a 32-electrodes belt 3D-EIT during SBT using	No statistically significant changes in EIT indexes measures before and during SBT	3D-EIT lung images feasible and more precise than 2D B
19	Wawrzeniak, [112]	ARDS mostly medical 25 (9)	44 (32)	10	Prospective Observational Single center	Transition from controlled mechanical ventilation to PS 8–12 cmH2O	Lung ventilation distribution across weaning categories (simple, difficult, prolonged)	Higher incidence of pendelluft, and redistribution of ventilation to posterior regions in the prolonged weaning category	Pathophysiological basis on the difference in weaning categories A

Definition of abbreviations: ICU intensive care unit, SBT spontaneous breathing trial, EIT electrical impedance tomography, GII global inhomogeneity index, RVD regional ventilation delay, PS pressure support, ATC automatic tube compensation, PEEP positive end expiratory pressure, CPAP continuous positive airway pressure, EELI end expiratory lung impedance, NA not applicable. The assessment of bedside availability of EIT indexes is made according to the following code: A. All indexes are available at the bedside, B most indexes are available at the bedside, C most indexes are not available at the bedside, D no index is available at the bedside

**Table 3:** Studies on electrical impedance tomography during chest physiotherapy in adult intubated ICU patients

# Study	First author, year (paper reference)	N Patients Case mix	Age, years (%male)	Mean days intubated	Study design	Respiratory physiotherapy technique	Primary end-points	Main results	Benefit of EIT assessment and bedside availability of EIT indexes
1	Linnane, [103]	9 Miscellaneous causes for intubation	59 (89)	3	Prospective, Randomised crossover Single center	Manual versus ventilation hyperinflation post- post-endotracheal suctioning	To determine which technique was better in restoring EELV	EELI decreased after endo-tracheal suctioning. Both techniques equally restored EELI above baseline	Monitoring EELI loss and management after end-tracheal suctioning A
2	Longhini, [100]	60 mostly medical	69 (58)	NA	Prospective Randomized Single center	High-frequency chest wall oscillation	Assessment of lung ventilation distribution after High-frequency chest wall oscillation in normosecretive and hypersecretive mechanically ventilated patients	EELI increased after High-frequency chest wall oscillation in hypersecretive patients	Help to select who will benefit from High-frequency chest wall oscillation A
3	Garofalo, [101]	15 Medical Tracheotomised	56 (53)	NA	Prospective Observational Single center	High-frequency percussive ventilation superimposed to mechanical ventilation	Lung ventilation distribution after High-frequency percussive ventilation	Increase in EELI (ventral and dorsal) and reduction in GII after High-frequency percussive ventilation	Monitor the effects of High-frequency percussive ventilation on lung ventilation A
4	Li, [95]	82 High‑dependency unit patients with pulmonary diseases	69 (71)	NA	Prospective Observational Single center	Postural drainage, assisted cough technique, positive expiratory pressure, high-frequency chest wall oscillation, chest percussion, vibration, and active cycle of breathing techniques based on EIT	Pilot study of EIT-guided chest physiotherapy in high‑dependency unit patients with pulmonary diseases	Significant improvement in ventilation distribution in all patients after the entire EIT-guided program	Feasibility of an EIT-guided program to optimize adjuncts of mechanical ventilation in ventilator-dependent patients A
5	Yildirim, [102]	20 Medical ARDS	64 (65)	NA	Prospective Randomized Single center	Open versus closed tracheal aspiration	Comparison of effects of methods to suction trachea on lung ventilation distribution	Drop in EELI after tracheal suction was reduced with the closed system	Assess the benefit of tracheal suctioning A
6	Sutt, [104]	20 tracheotomized	60 (50)	2.5	Prospective Observational Single center	Speaking valves	To assess the effect of speaking valves on EELI	EELI increased after the use of speaking valves	Monitor the effect of speaking valve on lung recruitment A
7	Sutt, [105]	20 tracheotomized	60 (50)	2.5	Prospective Observational Single center	Speaking valves	To assess whether speaking valves induce lung hyperinflation	Speaking valves do not promote lung hyperinflation	Monitor the effect of speaking valve on lung recruitment C

Definition of abbreviations: ICU intensive care unit, EIT electrical impedance tomography, NA not applicable, EELI end expiratory lung impedance, GII global inhomogeneity index, ARDS acute respiratory distress syndrome. The assessment of bedside availability of EIT indexes is made according to the following code: A. All indexes are available at the bedside, B most indexes are available at the bedside, C most indexes are not available at the bedside, D no index is available at the bedside

## Applications of EIT during patient body positioning

Technical issues should be kept in mind when EIT is used in different body positions. Special care should be required in order to ensure that the electrode belt position is not modified by the variation of body position to secure comparability of examinations performed in various body positions since the position of the electrodes may affect the EIT finding both in perfusion terms and in ventilation terms [2, 6, 23, 24]. Generally, a belt positioned between the 4th and 5th intercostal space is recommended even if the measurement conditions can modify this ideal belt position (e.g. wounded skin). Several other factors may impact the quality of the EIT measurement or the possibility to compare EIT measurements [6]. We can cite the number of electrodes, the quality of the electrode–skin contact, some pathologies as pneumothorax or pleural effusion, the presence of active pacemaker, the type of image reconstruction algorithm, the geometrical pattern used to represent the transversal section of the thorax with the lung and heart areas.

Table 1 summarizes the literature search on body positioning.

### Prone position

Prone position improves oxygenation in most patients, and, more importantly, makes lung aeration more homogeneously distributed, protecting the lung from excess in stress and strain, as compared to supine position [25]. EIT could improve this figure by deciphering the mechanisms of the oxygenation response to prone position and defining the optimal duration of the proning sessions based on lung ventilation distribution and ventilation/perfusion assessment [26]. When prone position is recommended for long sessions (at least 16continuous hours) [27] a more prolonged application has been advocated [28].

### Determinants of oxygenation improvement in prone position

A significant correlation was found in COVID-19 patients between a better oxygenation in prone and the amount of lung collapse in dorsal regions in supine position [29] and the improvement in ventilation-to-perfusion matching in prone [30, 31]. However, no relationship was found between better oxygenation in prone and greater lung homogeneity and better dorsal lung compliance in COVID-19 under assisted breathing [32].

### Redistribution of lung ventilation

In non-COVID-19 ARDS patients, lung compliance in the dorsal regions increased progressively over time in the prone position regardless of the PEEP strategy as compared to the supine position [33].

In 22 intubated COVID-19 related ARDS patients studied by Fossali et al. [34], the lung CT showed a net lung recruitment in prone as compared to supine position: the lung recruitment in the dorsal lung regions was greater than the lung derecruitment in the ventral lung regions in prone [34] in line with other studies [35]. The EIT findings followed the aeration data with a net higher lung ventilation in prone than in supine position: the increase in lung ventilation in the dorsal regions was greater than the reduction of lung ventilation in the ventral lung regions in prone position [34]. The lung ventilation redistribution towards non-dependent lung regions together with higher dorsal EELI in prone was also found in patient’s proned under ECMO [36]. However, as expected, this response can be different across patients [37]. In the study by Fossali et al., 2 in 22 patients did not follow the above scenario and did not improve aeration and ventilation in the dorsal lung regions in prone [34]. This heterogeneity was confirmed by Morais et al. in 22 patients intubated for COVID-19 related ARDS [38]: the above scenario of better lung ventilation in the dorsal lung regions in prone position was observed in a subgroup of patients whose respiratory system compliance increased. The decrease in collapse and overdistension in prone as compared to supine found with EIT confirms the lung protective nature of prone positioning [39]. New EIT approaches [40] may be useful to assess the lung protective nature of prone positioning. The increase in ventilation in the dorsal lung regions during invasive ventilation contrasted with no change during non-invasive ventilation in COVID-19 patients, for a similar improvement in oxygenation [41]. These findings may be explained by baseline difference in lung injury.

### EIT and personalization of PEEP titration in prone position

EIT can help titrating PEEP in the prone position based on the compromise between collapse and overdistension underscoring the need for individualized PEEP [42]. Above a PEEP of 10 cmH_2_O, prone position was associated with overdistension but less collapse than in the supine position [43]. The effects of pronation on lung ventilation are dependent on lung recruitability. In patients with high lung recruitability, dependent collapsed regions decreased at high PEEP in both supine and prone positions. In patients with low lung recruitability, low PEEP in prone position reduced dependent and non-dependent collapsed lung regions [44]. The role of EIT in the personalization of PEEP is still under investigation, and the effects of individualized PEEP remain uncertain.

### Effects of prone position on lung ventilation and perfusion assessed by EIT

EIT can not only measure lung ventilation but also lung perfusion [45], and hence ventilation-to-perfusion matching [46]. In COVID 19 patients, lung perfusion remained predominant in the dorsal lung in the prone position, with a decreased ventral dead space and dorsal shunt [47]. This was also found in non COVID ARDS [31]. A prospective study in two centers in 14 ARDS and 15 COVID intubated patients found slight differences between the two groups [48]. When proned, ventilation-to-perfusion matching improved in non-COVID ARDS, while it was overall reduced in COVID [48]. Distinct effects of pronation on ventilation-to-perfusion matching were observed according to the ARDS stage or ARDS phenotype. It improved in the early stage but worsened in the persistent stage [49]. For the ventilation-to-perfusion ratio to get improved a longer time in prone was required in diffuse ARDS or in ARDS with higher D-dimer blood concentration than in focal ARDS or in ARDS with lower blood D-dimer [50].

### EIT in awake prone position

The COVID-19 pandemic enhanced the use of awake prone positioning. In non-intubated patients with acute hypoxemic respiratory failure due to COVID-19 pneumonia treated by awake prone positioning, EELI consistently increased in this position [51, 52], while lung ventilation was unchanged [51] or shifted towards dorsal lung regions [52].

### Lateral position

The nondependent-to-dependent impedance ratio was investigated during lateral positioning in 33 patients with normal lung under general anaesthesia for elective urologic surgery and randomized into three groups of 11 patients each based on the level of PEEP: 0, 5, and 10 cmH_2_O [53]. The ratio increased in lateral position at PEEP 0 and 5 cmH_2_O but not at PEEP 10 cmH_2_O suggesting that PEEP 10 cmH_2_O redistributed the lung ventilation evenly across both lungs. Furthermore, the oxygen alveolar-arterial (A-a) gradient was better at PEEP 10 cmH_2_O than in the two other groups.

An early small study found no significant change in lung ventilation distribution in ARDS patients during continuous lateral positioning [108]. In another study in 5 COVID patients, the targeted lateral position, i.e.: the less aerated lung non-dependent and more aerated lung dependent, associated with EIT-guided PEEP, reduced overdistension and collapse [109]. Moreover, lateral position was found not to decrease lung volume in the dependent lung [110]. In intubated COVID patients, Roldan et al. found that the sequential lateral positioning improves lung ventilation distribution [54] (Table 1). In their study, this intervention increased respiratory system compliance and reduced driving pressure but also significantly improved PaO₂/FiO₂. Electrical impedance tomography revealed a marked increase in dorsal end-expiratory lung impedance, indicating effective dorsal recruitment, while lung ultrasound scores for juxta-pleural consolidation were reduced. Importantly, patients with a recruitment-to-inflation ratio above 0.5 experienced the greatest benefits, underscoring the value of targeted patient selection. The authors concluded that lateralization is a low-resource recruitment strategy that demands minimal staffing and is particularly useful when prone positioning is either contraindicated or difficult to implement [54]. Together, these findings suggest that EIT is helpful in guiding or selecting patients for sequential lateral positioning, although data from large scale studies are lacking.

### Semi-recumbent position

**Fig. 2:**
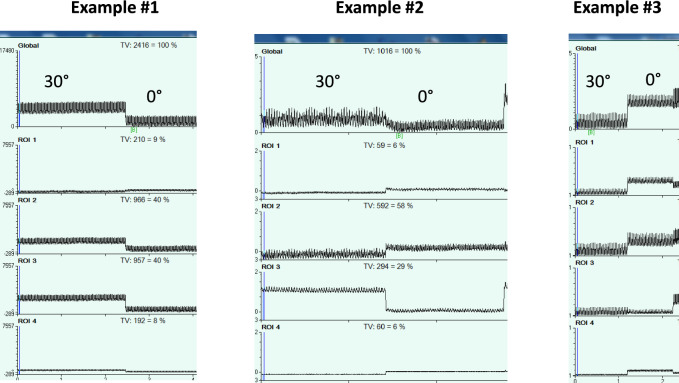
Unpublished personal data on the effect on impedance changes of changing body inclination from 30° to 0° in supine position in 3 patients with acute respiratory distress syndrome intubated and mechanically ventilated in volume control mode with 6 ml/kg predicted body weight tidal volume, positive end-expiratory pressure of 5 cmH2O, not individualized. The body mass index was 23, 32 and 18 kg/m2, in examples 1, 2 and 3, respectively. In examples #1 and #2 global end-expiratory lung impedance (EELI) went down when inclination moved from 30° to 0°. In example #1 this resulted from the decrease of EELI in all the regions of interest (ROI) but the fourth (most dependent). In example #2 the magnitude of EELI decrease in the ROI 3 was greater than the magnitude of EELI increase in the three other ROIs. In example #3 by contrast, global EELI increased when inclination moved from 30° to 0° as a result of an EELI increase in the ROIs 1 and 2. These findings illustrate why EIT monitoring can be useful to select an optimal angulation of the body in a given patient

The semi-recumbent position is the recommended body position in intubated ICU patients in general. However, the optimal angulation of the body can differ across patients depending on how much lung and chest wall mechanics are affected. EIT can be useful to define an optimal body angulation promoting a greater lung ventilation homogeneity. A semi-recumbent position is generally recommended. The reasons for this include higher end-expiratory lung volume [55] and lower incidence of ventilator-associated pneumonia [56] compared to the supine position. However, the overall consequences of changing the trunk inclination depend on the respective effects on the lung and chest wall compliance [57] and the elevation of the diaphragm towards the cranial direction due to a reduction in lung volume [58]. Moreover, progressive trunk inclination increases pulmonary vascular resistance and hence may affect lung perfusion distribution [59]. Therefore, in this setting EIT can be useful to define the optimal trunk inclination. The response to body trunk inclination in terms of EELI in ARDS patients can be dramatically different despite a same level of PEEP. Examples of this variable response are shown in Fig. 2. While the global EELI decreases in example #1, it changes minimally or increases in examples #2 and #3, respectively. This suggests the utility of EIT for personalizing semi-recumbent positioning in this patient population.

Normal volunteers breathing spontaneously were investigated in the supine position followed by a bed inclination of 30, 60 and 90° [60]. While no significant effect of position change was observed in EIT indexes, the variability across subjects was wide. The impact of the head-up position was tested during cardiac resuscitation in human cadavers [61]. With the head-up position, the EELI was found consistently reduced by the chest compressions while the other EIT parameters did not change [61].

In intubated ICU patients (Table 1), Chen et al. found that change in EELI decreased in ventral and increased in dorsal parts of the lungs at 70° angulation or at sitting position as compared to the prior supine position in intubated ARDS patients [62]. Moreover, the dorsal and ventral lung compliances remained higher when returning back to supine position suggesting that the sitting position promoted overall lung recruitment [62]. In COVID-19 patients, semi-recumbent position was associated with a better lung ventilation homogeneity than supine position [63].

## Applications of EIT during weaning

The weaning process in intubated patients includes the following steps: detection of weanibility, testing weanibility through a spontaneous breathing trial (SBT), extubation of those who succeed the SBT [64] and securing the post-extubation period in patients at risk of extubation failure [65]. Weaning failure, defined by death or required invasive ventilation at 90 days, occurred in 15.6% of patients, and delayed onset of weaning was associated with a higher risk of weaning failure [66]. The pathophysiological mechanisms subtending weaning failure are complex [67]. EIT can be useful to address the following issues: is loss of lung volume greater in weaning failure than in weaning success? Which among SBT techniques [6] better prevents loss of lung volume over time? Is heterogeneity of lung ventilation before weaning attempt associated to weaning failure? Can PEEP level tailored according to intensity of inspiratory effort improve ventilation distribution? EIT indexes previously discussed can provide insight into these issues.

The patient’s respiratory effort is intrinsically linked to the weaning process and, combined with the severity of lung injury, is a key factor of weaning outcome. On one hand, a gentle effort applied to a mild-moderate lung injury can have positive effects in promoting lung recruitment and increased ventilation in the dorsal regions due to diaphragm activity [7, 68]. On the other hand a strong patient effort superimposed on a severe lung injury may have a negative effect depicted as patient-self-inflicted injury [69]. This lung injury results not only in a higher global lung stress (excessive end-inspiratory trans-pulmonary pressure) but also in a higher local stress in the dependent lung regions [70]. Part of this nondependent lung stretch in the dependent regions is due to the pendelluft phenomenon. EIT is a primary tool to investigate the interaction between the strength of patient effort and the lung ventilation distribution. Notably, EIT is currently the only bedside method available to detect the pendelluft phenomenon [12, 71]. In this respect, the increased distribution of ventilation in the dorsal lung regions may lead to misinterpretation of EIT signals in the presence of pendelluft. Taking into account the phase of the breathing cycle avoids this confusion between lung recruitment and air redistribution. Using EIT, a 31% prevalence of pendelluft was found in intubated and mechanically ventilated ICU patients which was associated with longer mechanical ventilation length [72]. Pendelluft was observed in 50% of spontaneously breathing ARDS patients [73] and in 70.4% of difficult to wean patients during SBT. Also, it has been associated with a higher mortality at day 28 [74]. EIT assessment of pendelluft together with monitoring of breathing effort might help providing a safer ventilatory assistance [75].

### Setting the ventilator in spontaneously assisted breathing patients

The transition phase from controlled to spontaneous assisted breathing is associated with a risk of lung injury as discussed above. Therefore, setting the ventilator should be as meticulous as it is the early phase of passive mechanical ventilation. In ARDS patients breathing spontaneously, the effort-related injurious lung stretch in the dependent regions can be attenuated by higher PEEP [70]. Monitoring EELI during a decremental PEEP trial in ARDS patients has been shown to enable determining the level of PEEP that maintains lung recruitment based on the stability of the EELI signal [76]. Mauri et al. recently reported a crossover trial in 30 ARDS patients breathing spontaneously in PS [77]. A personalized PEEP selected using EIT and trans-pulmonary pressure minimized overdistension and collapse compared to a control group (PEEP/FIO_2_ table). In the experimental group, PEEP (10 cmH_2_O vs. 8 cmH_2_O in the control group) was associated with reduced dynamic lung stress and metabolic work of breathing [77]. On the other hand, Bello et al. monitored the regional respiratory compliance changes with EIT in 16 patients with moderate-to-severe ARDS exhibiting intense inspiratory effort on assisted ventilation [78]. A high PEEP of 15 cm H_2_O had a highly variable effect on inspiratory effort, which depended on the induced change in overall EIT-measured regional compliance: the subjects with an increased compliance had a reduced inspiratory effort [78]. This data suggests that EIT may guide the selection of the appropriate levels of PEEP when an intense inspiratory effort is present.

In a study in 20 patients, Bassi et al. pendelluft was common in patients with acute hypoxemic respiratory failure on spontaneous assist-breathing [73]. The magnitude of pendelluft was higher with higher respiratory elastance and inspiratory effort, and the response to PEEP variable [73]. A higher incidence of pendelluft together with greater redistribution of ventilation towards posterior regions has been observed in prolonged weaning compared to simple or difficult weaning [76].

### EIT-assessment of spontaneous breathing trials

Table 2 summarizes the role of EIT in the assessment of SBT. SBT failure is expected to be associated with lower EELI, higher GII, and higher pendelluft as compared to SBT success. These hypotheses were verified in several studies. GII was higher in SBT failure than SBT success in 4 studies [79–83], with average values of area under curve (AUC) for GII to predict SBT failure of 0.81 [79], 0.73 [80], 0.74 [81] during SBT, and 0.69 before SBT [83]. In patients who successfully passed an SBT, GII was higher in those who eventually failed extubation with an AUC of 0.76 and 0.63 at two hours and six hours after extubation, respectively [84]. Recently, the prediction of the absolute ventral-to-dorsal difference of the change in lung impedance had a good prediction of SBT failure with an AUC of 0.82 and 0.84 at 2 and 5 min after SBT onset, respectively [85]. For the EELI or its change during SBT, its performance to predict SBT failure was excellent in one study with an AUC of 0.91 and 0.95 at 10 and 30 min after SBT onset [86]. Same findings were observed in a retrospective study in critically ill after abdominal surgery [87]. The performance of EELI was moderate in other investigations with an AUC of 0.77 at onset and 30 min of SBT [81] or 0.72 at 20 min of SBT [79]. In the latter study TSA had a 0.94 AUC to predict SBT failure [79]. When pressure support (PS) level was lowered to 2 cmH2O pendelluft increased regularly in particular in the dorsal lung regions and was associated with increased end tidal CO2 [8]. During SBT, total and ventral pendelluft had a 0.75 and 0.76 AUC, respectively, to predict SBT failure at 10 min of SBT but AUC went down to 0.66 when pendelluft was added to the absolute ventral-to-dorsal difference of the change in lung impedance [85]. This study also found that the ventilation distribution patterns during the weaning phase can predict worse outcomes [85]. This aligns with previous findings by Iwata et al. [88], who used ventilation distribution phenotypes to predict postoperative pulmonary complications.

**Fig. 3:**
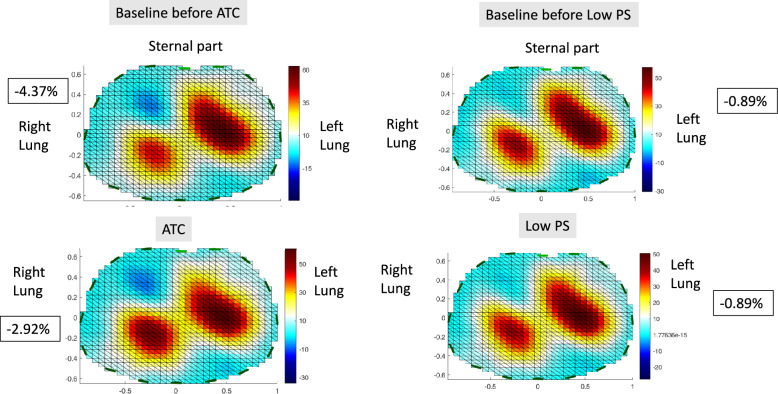
Lung ventilation assessed by electrical impedance tomography in one patient taken from reference [90] before (baseline) and during either automatic tube compensation (ATC) or pressure support (PS). At baseline, the ventilator was set in PS 12 cmH_2_O and PEEP 5 cmH_2_O. ATC was set at 100% inspiratory tube compensation, 0% expiratory tube compensation, positive end-expiratory pressure (PEEP) 4 cmH_2_O and PS 0 cmH_2_O. Low PS was set at 7 cmH_2_O and PEEP 4 cmH_2_O. The blue color code indicates pendelluft, whose values expressed in percent tidal volume are shown into the black squares. The EIT signals were processed with the EIT reconstruction software EIDORS using a GREIT model [106]

EIT indexes were measured in studies comparing SBT techniques. Zhao et al. compared automatic tube compensation (ATC) and continuous positive airway pressure (CPAP), each at two levels, and found that the higher level was associated with a higher EELI and a redistribution of intra-tidal gas towards dorsal regions [89]. In a study comparing ATC to low pressure support (PS), when higher breathing power was found in the ATC group, the lung ventilation distribution was not different between ATC and low PS [90] (Fig. 3).

Bosch-Comte et al. compared T-Piece versus low PS and found that EELI decreased during SBT with no difference between the SBT modalities [91]. Wang et al. also did not find a difference in any EIT index including pendelluft between T-piece, ATC and CPAP as SBT modalities [92]. As the various EIT indexes markedly changed from patient to patient, the authors pointed out that different EIT indexes should be selected in specific patients, suggesting a personalised approach of EIT during the weaning. Coudroy et al. studied the change in EELI at the end of a SBT in 25 patients enrolled in a large multicentre trial comparing T-piece and low PS [93]. EELI decreased in both modalities but the magnitude was higher with T-piece than low PS and returned to the pre-SBT value once mechanical ventilation was resumed [93].

The EIT assessment of SBT may benefit from technological advances with the use of two rows of 16 electrodes using a simultaneous multicurrent source EIT system for 3D imaging of the lung [94].

## Applications of EIT during chest physiotherapy

Chest physiotherapy includes different components in ICU patients: clearing airway secretions, avoiding lung derecruitment, active mobilization, exercising, improving speech and communication. EIT can guide this management [95]. In intubated ICU patients secretion accumulation is an important cause of extubation failure [96]. Therefore, an efficient clearing of airway secretions is important [71]. Mechanical insufflation-exsufflation (MIE) can be used to achieve this task [97] among other techniques [98] . By assessing the effect of MIE devices on lung ventilation distribution the above discussed EIT indexes can guide the management of clearing airway secretions.

EIT can be useful to assess the procedure [99] (Table 3). In 60 ICU patients under invasive ventilation Longhini et al. found that high-frequency chest wall oscillation increased EELI change and dorsal lung ventilation whether or not they were normo- or hypersecretive or received a recruitment manoeuvre [100]. Garofalo et al. investigated High-frequency percussive ventilation superimposed to mechanical ventilation and found increase in ventral and dorsal EELI and reduction in GII after the procedure [101]. The drop in EELI after tracheal suction was reduced with a closed system as compared with an open one [102] when both manual ventilation of hyperinsufflation equally restored EELI [103].

In tracheotomised ICU patients every effort to promote speech and communication is important for improving the quality of life of the patients. Sutt et al. found that a speaking valve used at the time of weaning from mechanical ventilation in 20 tracheotomised ICU patients improved EELI, meaning that not only does it facilitate speech and communication with relatives and caregivers but also it recruits some parts of the lungs [104]. A further study by the same investigators found that the speaking valve did not promote hyperinflation [105].

**Fig. 4:**
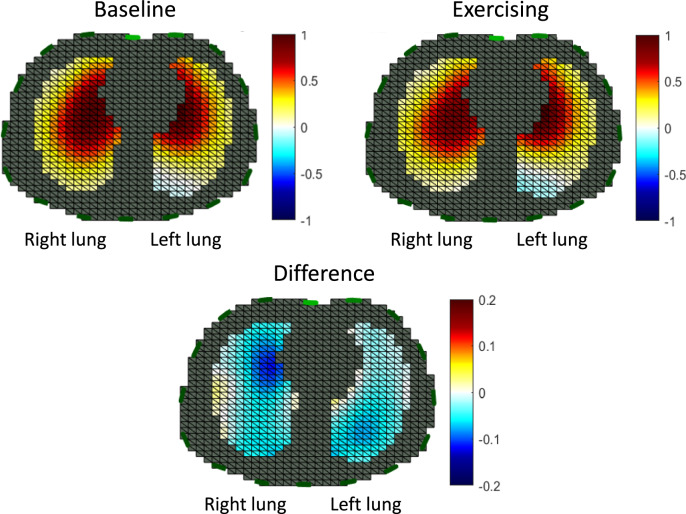
Unpublished personal data of the lung ventilation distribution assessed by electrical impedance tomography in an intubated patient under assisted-volume controlled ventilation in two conditions. On the top left panel the patient is resting in the semi-recumbent position in his bed. The top right panel shows distribution of ventilation during the last 2 min after exercising on a cyclo-ergometer for 10 min in the semi-recumbent position in his bed. The bottom panel shows the difference between the two conditions. Between baseline and exercising the antero-posterior distribution of the impedance was 1.26 and 1.48, the center of ventilation 48.2 and 46.5% and the pendelluft (indicated as blue in the color code) − 0.43 and − 2.13% of tidal volume, respectively. The EIT signals were processed with the EIT reconstruction software EIDORS using a GREIT model [106]

It should be noted as a limitation that performing EIT assessment during chest physiotherapy can generate noise in impedance signal as observed in cadavers during chest compressions [61]. Finally, EIT could also be used to assess the potential changes in lung ventilation distribution during active rehabilitation of critically ill patients like exercising under assisted spontaneous breathing assistance (Fig. 4).

## Conclusions

EIT offers the opportunity to monitor lung ventilation and lung perfusion at the bedside and to deliver ventilator strategies taking into account the patient’s physiologic condition, hence enabling a personalized management. During body positioning, EIT helps identify how ventilation and perfusion redistribute with prone, lateral, and semi-recumbent positions. It enables individualized assessment of PEEP response and recruitability, detects heterogeneity in patient response to prone positioning, and allows monitoring the use of lateralization as an alternative when prone positioning is not feasible. In the weaning process, EIT offers unique capabilities to assess lung derecruitment (via EELI), ventilation heterogeneity (via GII), and pendelluft, which are critical to predicting spontaneous breathing trial (SBT) success or failure. It also allows for real-time adjustment of ventilator settings to minimize patient self-inflicted lung injury and tailor PEEP during assisted breathing. During chest physiotherapy, EIT supports evaluation of secretion clearance techniques, recruitment maneuvers, and interventions like speaking valves by visualizing regional ventilation changes and confirming effectiveness. Across all these applications, the available evidence relies on small studies with a limited number of enrolled patients. Further well designed large-scale EIT studies should be performed during from mechanical ventilation to assess if the technique is useful at this stage. Further EIT studies should also been done to better understand the effects of changing body position and chest physiotherapy on lung ventilation and perfusion distribution in ICU patients.

## Supplementary Information

Additional file 1

Additional file 2

## Data Availability

Not applicable.

## Abbreviations

ARDS: Acute respiratory distress syndrome
ATC: Automatic tube compensation
AUC: Area under the curve of the receiver operating characteristic
CPAP: Continuous positive airway pressure
CT: Computed tomography
ECMO: Extracorporeal membrane oxygenation
EELI: End expiratory lung impedance
EIT: Electrical impedance tomography
GII: Global inhomogeneity index
ICU: Intensive care unit
PEEP: Positive end expiratory pressure
PS: Pressure support ventilation
ROI: Region of interest
SBT: Spontaneous breathing trial
TSA: Temporal skew of aeration
VTEIT: Lung ventilation
