# Supplementary material for: Lung electrical impedance tomography during positioning, weaning and chest physiotherapy in mechanically ventilated critically ill patients: a narrative review
Source: Ann Intensive Care. 2025 Aug 29;15:127. doi: 10.1186/s13613-025-01526-z (PMC12394117; doi:10.1186/s13613-025-01526-z)
Supplement: Supplementary file 1 — Additional file 1 [file 13613_2025_1526_MOESM1_ESM.docx]

Additional table 1. Short description of EIT indexes available at the bedside

| EIT index available at the bedside | Short description |
| --- | --- |
| Ventilation distribution | Change in pixel impedance between end-expiration and end-inspiration (ΔZ) over the whole lung or in some regions of interest (ROI) arranged in 4 layers, or pertaining to ventral and dorsal lung, or to right and left lung, or to dependent or non-dependent parts of the lung. In each ROI the regional ventilation (VTreg) is normalized by the total VT (ΔZ sum of whole lung) |
| End-expiratory lung impedance (EELI) | The minimal value of ΔZ sum in each cycle. Can be inferred over the whole lung or in each ROI. |
| Dorsal fraction of ventilation | Computed as 100 x (ΔZ sum of the dorsal lung)/ (ΔZ sum of the whole lung). A value of 50% means that the ventilation is homogenously distributed along the ventral-to-dorsal gradient. The same computation is also named coefficient of variation. |
| Center of ventilation | Computed as the ratio between i) the ΔZ weighted by the vertical position sum and ii) ΔZ sum. Conceptually relatively similar to the center of gravity. |
| Overdistension and collapse | Based on the measurement of regional compliance (VTreg/ΔP) and the observation of an increase and then a decrease in regional compliance with positive end expiratory pressure (PEEP) progressive reduction, corresponding to overdistension and lung collapse, respectively. Requires a decremental PEEP trial after a recruitment manoeuver. |
| Regional ventilation delay | Time delay between the onset of mechanical inspiration and the reaching of a specific impedance threshold. Requires a low flow insufflation |
| Silent spaces | Computed as the ratio between i) the number of pixels with a ΔZ < 10% to maximal change of pixel impedance and ii) the total number of pixels (% of unit lung) |
