# Supplementary material for: Lung electrical impedance tomography during positioning, weaning and chest physiotherapy in mechanically ventilated critically ill patients: a narrative review
Source: Ann Intensive Care. 2025 Aug 29;15:127. doi: 10.1186/s13613-025-01526-z (PMC12394117; doi:10.1186/s13613-025-01526-z)
Supplement: Supplementary file 2 — Additional file 2 [file 13613_2025_1526_MOESM2_ESM.docx]

Additional file 2. Literature search strategy

One of us (CG) searched on PubMed from inception to 22 May 2025 original articles based on the following inclusion criteria: adulthood (≥18 years old), male or female, intubated for acute respiratory failure and mechanically ventilated in intensive care units, prospective or retrospective observational clinical studies or randomized controlled trials, English language.

Filters were used to exclude studies if they reported less than 5 cases, were reviews, systematic reviews, animals studies, studies in children (18 years old), in abstract format only, and without data (study protocol).

The following terms were searched in all fields.

| Search | terms |
| --- | --- |
| #1 | Lung AND electrical impedance AND tomography |
| #2 | #1 AND mechanical ventilation |
| #3 | #2 AND positioning |
| #4 | #3 AND prone |
| #5 | #3 AND lateral |
| #6 | #3 AND semi recumbent |
| #7 | #2 AND weaning |
| #8 | #2 AND spontaneous breathing trial |
| #9 | #7 AND positive end expiratory pressure |
| #10 | #7 AND spontaneous breathing trial |
| #11 | #2 AND physical therapy |
| #12 | #2 AND physiotherapy |

Papers retrieved from the search strategy were further excluded after reading the title, then after reading the abstract. Additional papers may be retrieved from the reading of the list of the references in the selected papers (snowballing). The searches #1, #2 and #3 are common to the three sections. The searches #4 to #12 were then split into the three main sections of the review with their corresponding tables.

| Search | Articles retrieved | Articles selected after reading title | Articles selected after reading abstract | Final number of papers in the tables selected + snowballing | Number of the tables in the review | Section of the review |
| --- | --- | --- | --- | --- | --- | --- |
| #4 | 59 | 13 | 13 | 25 | 1 | Positioning |
| #5 | 21 | 5 | 4 |  |  |  |
| #6 | 1 | 1 | 1 |  |  |  |
| #7 | 49 | 18 | 11 | 19 | 2 | Weaning |
| #8 | 30 | 19 | 11 |  |  |  |
| #9 | 20 | 7 | 5 |  |  |  |
| #10 | 17 | 10 | 6 |  |  |  |
| #11 | 14 | 3 | 1 | 7 | 3 | Chest physiotherapy |
| #12 | 27 | 8 | 6 |  |  |  |
